# Morphological Study of Nanostructures Induced by Direct Femtosecond Laser Ablation on Diamond

**DOI:** 10.3390/mi12050583

**Published:** 2021-05-20

**Authors:** Ahmed Abdelmalek, Argyro N. Giakoumaki, Vibhav Bharadwaj, Belén Sotillo, Thien Le Phu, Monica Bollani, Zeyneb Bedrane, Roberta Ramponi, Shane M. Eaton, Malik Maaza

**Affiliations:** 1 Physics Department, Theoretical Physics Laboratory, Tlemcen University, Tlemcen 13000, Algeria; ahmed7abdelmalek13@gmail.com (A.A.); zeyneb_bedrane@yahoo.fr (Z.B.); 2 Department of Physics, Politecnico di Milano, Piazza Leonardo da Vinci, 32, 20133 Milano, Italy; argyrogiak@gmail.com (A.N.G.); thien.lephu@polimi.it (T.L.P.); roberta.ramponi@polimi.it (R.R.); 3 Institute for Photonics and Nanotechnologies—CNR-IFN, Piazza Leonardo da Vinci, 32, 20133 Milano, Italy; monica.bollani@ifn.cnr.it (M.B.); shane.eaton@gmail.com (S.M.E.); 4 Materials Physics Department, Faculty of Physics, Complutense University of Madrid, 28040 Madrid, Spain; bsotillo@gmail.com; 5 UNESCO-UNISA Africa Chair in Nanoscience and Nanotechnology, College of Graduate Studies, University of South Africa, Muckleneuk Ridge, P.O. Box 392, Pretoria 0001, South Africa; maaza@tlabs.ac.za; 6 Nanosciences African Network (NANOAFNET), iThemba LABS-National Research Foundation, 1 Old Faure Road, P.O. Box 722, Somerset West, Western Cape 7129, South Africa

**Keywords:** femtosecond laser, LIPSS morphology, 2D-FFT, plasmonic excitation

## Abstract

High spatial frequency laser induced periodic surface structure (HSFL) morphology induced by femtosecond laser with 230 fs pulse duration, 250 kHz repetition rate at 1030 nm wavelength on CVD diamond surface is investigated and discussed. The spatial modification was characterized and analyzed by Scanning Electron Microscopy (SEM), Atomic Force Microscopy (AFM) and 2D-Fast Fourier Transform (2D-FFT). We studied the effect of pulse number and laser power on the spatial development of nanostructures, and also deduced the impact of thermal accumulation effect on their morphology. A generalized plasmonic model has been used to follow the optical evolution of the irradiated surface and to determine the periodic value of the nanostructures. We suggest that non-thermal melting and plasmonic excitation are the main processes responsible for the formation of HSFL-type nanostructures.

## 1. Introduction

Material modification by femtosecond laser pulses is a burgeoning field in both academic [1] and industrial [2] settings, thanks to minimal thermal effects compared to longer pulse durations [3]. Laser induced periodic surface structure (LIPSS) is a universal phenomenon that occurs on the surface of materials during laser—matter interaction [4]. For a normal incidence laser beam, we can distinguish two types of LIPSS according to their spatial periodicity, low spatial frequency LIPSS (LSFL) with period Λ>λ/2 and high spatial frequency LIPSS (HSFL) with period Λ<λ/2. Each of these types contains two other forms depending on the wavevector of the gratings with respect to the polarization of the incident beam; ${\mathrm{LSFL}}_{∥,⊥}$ and ${\mathrm{HSFL}}_{∥,⊥}$ parallel or perpendicular to the incident laser polarization [4].

In diamond, Wu et al. [5] highlighted the presence of three types of LIPSS on the surface after femtosecond (fs) laser irradiation: ${\mathrm{LSFL}}_{⊥}$ with a periodicity dependent on the angle of incidence, ${\mathrm{HSFL}}_{⊥}$ with periodicity less than λ/3 and independent of the angle of incidence and ${\mathrm{HSFL}}_{∥}$ with a constant periodicity of some tens of nanometers, independent of the angle of incidence. Both ${\mathrm{LSFL}}_{⊥}$ and ${\mathrm{HSFL}}_{⊥}$ were observed on the diamond surface under fs laser irradiation, with ${\mathrm{LSFL}}_{⊥}$ being the dominant characteristic at higher fluences and a lower number of pulses, while the formation of ${\mathrm{HSFL}}_{⊥}$ favored at low fluence and high number of pulses [6]. Apostolova et al. [7] observed that the two types of LIPSS, ${\mathrm{LSFL}}_{⊥}$ and ${\mathrm{HSFL}}_{⊥}$ are dependent on the number of pulses. It is observed that ${\mathrm{LSFL}}_{⊥}$ and ${\mathrm{HSFL}}_{∥}$ are always perpendicular to each other [8]. ${\mathrm{LSFL}}_{⊥}$ depends on the number of pulses, fluence and wavelength while ${\mathrm{HSFL}}_{∥}$ does not depend on wavelength and it has a weak dependence on the number of pulses [9]. In our previous work [10], we showed that the ${\mathrm{HSFL}}_{⊥}$ type is influenced by the number of pulses and the incident laser wavelength and it has a low dependence on fluence. Due to the different dependencies of the parameters for each type of LIPSS, we suggest that the physical origin of each type is necessarily different.

It is widely accepted in the scientific community that the origin of LSFL-type nanostructures is due to the interference between the Surface Plasmon Polariton (SPP) and the incident laser beam, resulting in spatially modulated energy deposition on the material surface [11,12,13,14]. The origins of the HSFL type, in its two forms, is still under discussion, and is often explained by the second harmonic generation [15,16] or the plasmonic excitation [17,18] model.

Diamond is the hardest naturally occurring material, it has a record high thermal conductivity and offers excellent transparency from ultraviolet to far infrared. Femtosecond laser micromachining has opened opportunities for bulk and surface modification of diamond. Bulk modification of diamond has been applied for the formation of integrated photonics such as Type II waveguides and color centers for quantum information technologies [19,20,21]. Diamond surface micromachining has demonstrated that it is possible to create LIPSS based micro solar cells due to the formation of “black diamond” [22]. This type of nanostructure exhibits efficient optical response to THz radiation [23].

In this work, we fabricated nanostructures on the surface of optical grade synthetic diamond by focused femtosecond laser pulses. We studied the morphology of high spatial frequency laser induced periodic surface structure (HSFL) by varying the laser writing parameters such as the impact of number of pulses and fluence. The surface modifications are characterized by Scanning Electron Microscopy (SEM) and Atomic Force Microscopy (AFM). Our results show that HSFL morphology is strongly dependent on laser writing parameters. The generalized plasmonic model allowed us to understand the optical development of the surface during irradiation, as well as to calculate the periods of the induced nanostructures. We propose a theory for a physical description of the fundamental origin of the formation of ${\mathrm{HSFL}}_{⊥}$-type nanostructures. We suggest that we can improve the optical properties of the modified surface based on this kind of LIPSS if we adjust the laser parameters to get more precise structures.

## 2. Experimental Setup

**Figure 1:**
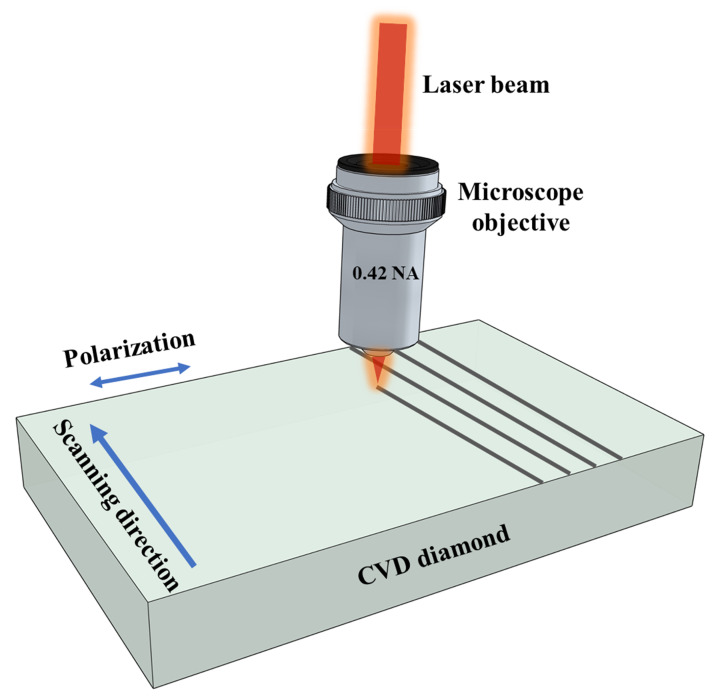
Schematic layout of the femtosecond laser writing setup.

A direct femtosecond laser micro-nanomachining workstation was used for nanotexturing of diamond surfaces. The samples used in this work were polished single crystal CVD diamond (MB Optics) with dimensions 7.5 mm × 7.5 mm × 0.5 mm. The initial surface roughness was measured to be less than 1 nm. An amplified femtosecond laser (Light Conversion Pharos) delivering 230 fs pulses at 1030 nm wavelength at a repetition rate of 250 kHz was used to irradiate the samples. The laser was incident normal to the diamond surface and focused with a 0.42 NA (50×) microscope objective, with a spot size of approximately 1.6 μm. The diamond sample was translated with respect to the focus using high resolution 3-axis computer controlled motion stages (ABL1000, Aerotech, Pittsburgh, PA, USA) to write straight 1-mm long lines on the surface. The incident average laser power was varied using a combination of a Glan laser polarizer preceded by a half waveplate on a computer-controlled rotation stage. The power stability of the laser is <0.5% of the R.M.S power value for our laser system. The number of laser pulses spatially overlapped within a laser spot size during the scan, *N* was varied by changing the scanning speed *v*, by following the relation: $N=(2\omega_{0}f_{r})/v$ where $f_{r}$ is the repetition rate and $2\omega_{0}$ is the laser spot size, where $\omega_{0}=\frac{\lambda }{\pi \mathrm{NA}}$ and λ is laser wavelength. The laser polarization was perpendicular to the scanning direction, and all the experiments were carried out in air. The layout of the experimental setup is shown in Figure 1.

The morphological characterization of the samples was performed ex situ by scanning electron microscope (Philips XL30 SFEG SEM, Oberkochen, Baden Württemberg, Germany) imaging, while AFM topography images have been acquired in tapping mode with a Veeco Innova instrument. Super-sharp silicon AFM probes (typical radius of curvature 2 nm) have been used for high-resolution imaging of nano-roughness, while standard silicon probes (15 nm as radius of curvature) have been employed for large-area scans in order to evaluate the flat diamond area. Free and open source software (Gwyddion) was used to perform 2D-Fast Fourier Transform (2D-FFT) of the SEM images.

## 3. Results and Discussion

### 3.1. Experimental Results

**Figure 2:**
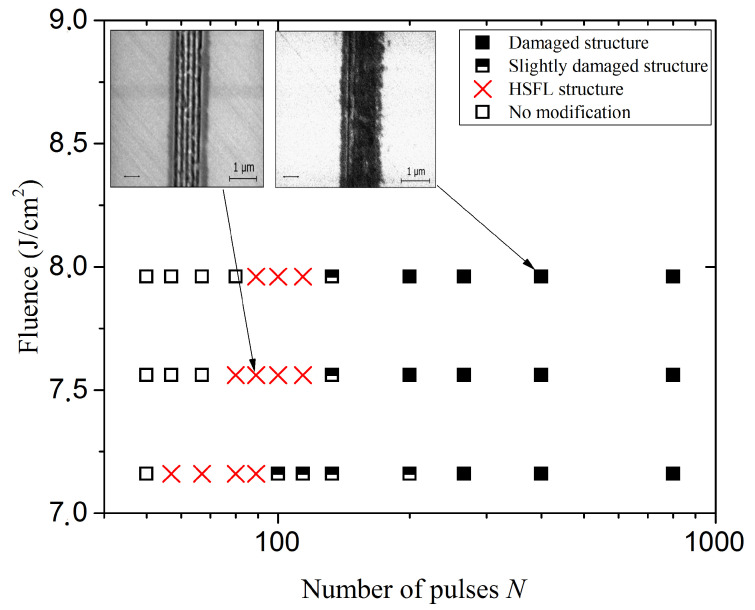
Summary of the modification results obtained during diamond surface irradiation by multipulse femtosecond laser for varying laser fluence and number of pulses.

In order to study the effect of laser parameters on the morphology of HSFL type nanostructures, the diamond surface was irradiated with the femtosecond laser where the pulse number was varied from 50 to 800 pulses per laser spot (*v* = 0.5–8 mm/s) for average laser powers of *P* = 18, 19 and 20 mW corresponding to fluence *F* = 7.16, 7.56 and 7.96 $J/{\mathrm{cm}}^{2}$. These average powers were chosen to be near the damage threshold of diamond, where nanostructures may be observed. The resulting structures were observed under SEM. Figure 2 summarizes the results of the surface modification, where three main regions can be distinguished; (1) damaged and slightly damaged structure, (2) HSFL structure and (3) no modification of the surface.

From preliminary analysis, we note from Figure 2 that at low number of pulses there is no modification at the surface, which confirms that nonlinear processes, such as multiphoton ionization, are necessary for each structuring in wide bandgap material. On the other hand, with a large number of pulses, we observe an ablation and a damage of the nanostructures where we suggest that it strongly returns to the effect of thermal accumulation.

Under our experimental conditions, we observed a narrow window of the appearance of nanostructures between no modification and damage, which we attribute to the mechanisms for the formation of nanostructures on the surface of dielectric materials.

If the number of pulses is low (high scanning speed), the density of electrons excited should be insufficient to form a pseudo-metal layer, leading to an absence of morphological modification on the surface. Conversely, if the number of pulses is high (low scanning speed), the density of the free electrons excited increases with pulse number until the saturation is reached, in which case the laser energy of the following pulses will be absorbed directly by the free electrons as if the material is a metal and transfer their energy to the bulk via electron–phonon collision. The repetition of this process after each pulse leads to the phenomenon of thermal accumulation effect, leading to the ablation of the material while it is a pseudo-metal. More explanation of the original mechanism of nanostructure formation is discussed in the following sections.

#### 3.1.1. Effect of Pulse Number

**Figure 3:**
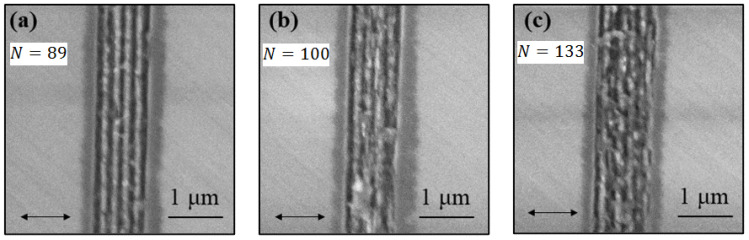

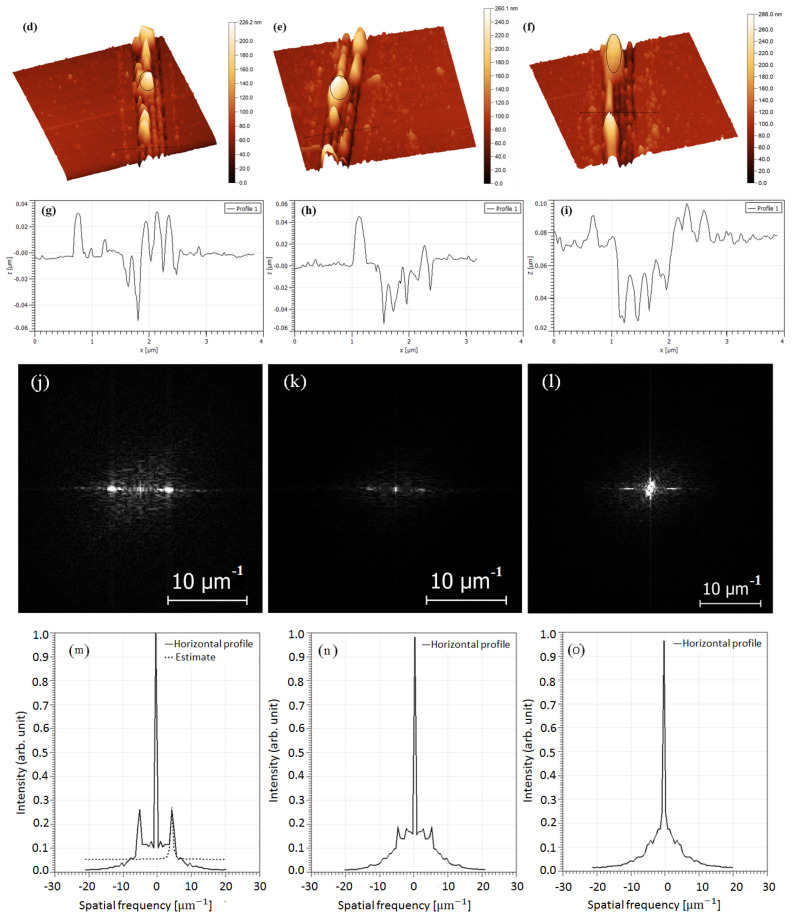
Representation of effect of pulse number on the morphology of the diamond surface irradiated by fs laser pulses with power of *P* = 19 mW ($F=7.56\;J/{\mathrm{cm}}^{2}$). (**a**–**c**) shows planar view SEM images of structures induced by *N* = 89, 100 and 133 pulses. (**d**–**f**) AFM measurements recorded in tapping mode of the (**a**–**c**), respectively (measured area 5 × 5 ${\mu m}^{2}$). (**g**–**i**) represent the cross-section profile corresponding to the lines marked in (**d**–**f**), respectively. (**j**–**l**) represent 2D-fast Fourier transforms (2D-FFT) and (**m**–**o**) its cross section horizontal profiles, respectively. The double-arrow shows the polarization of the incident laser beam.

Figure 3 shows the effect of pulse number on the morphology and the spatial evolution of HSFL under the experimental conditions used. Figure 3a–c shows SEM images of the diamond surface irradiated by a fs laser with a scanning speed 4.5, 4 and 3 mm/s (*N* = 89, 100, 133), with incident laser power *P* = 19 mW. We observe the formation of nanostructures of HSFL type perpendicular to the laser polarization along microgroove written by laser. In addition, the surface is less damaged when the scanning speed is high, where the formation of LIPSS is more regular as seen in Figure 3a which is formed by 89 pulses. As the number of pulses increases, more material is removed, as can be seen in Figure 3b,c. Figure 3d–f shows the AFM corresponding to Figure 3a–c, respectively. This 3D image shows that the LIPSS has multi-nano-walls inside the microgrooves along the laser writing. The circles mentioned in the 3D figures represent the regions of LIPSS damaged due to the thermal accumulation effect. Consequently, to obtain well regular nanostructures it is necessary to minimize the thermal effect. Because the energy absorbed by the free electrons can be transferred to the bulk by electron–phonon collision, the heat residue after each pulse leads to the phenomenon of thermal accumulation. Therefore, the thermal accumulation effect influences the morphology of LIPSS and directly affects the regularity of nanostructures.

Figure 3g–i represents a crater cross section profile corresponding to Figure 3a–c, respectively. The cross-section of the microgrooves gives more information on the structure of LIPSS. We observe that the height of nano-walls relative to the bottom of microgroove is reduced when the number of pulses increases. Consequently, the ablated volume of microgroove increases.

We notice that the depth of microgroove is around 60 nm, this value represents the thickness of the induced graphite layer during irradiation, and it well confirmed by our plasmonic model below.

Under multi-photoionization a pseudo-metallic layer (graphitic layer) is built up due to the phenomenon of disorder of the material [10,18]. Therefore, the origin of micro-grooves is due to the phase explosion due to the thermal accumulation effect induced during multipulse laser irradiation [24]. However, the ablation of the diamond surface by a single pulse is mainly due to the Coulomb Explosion (CE) [25]. Thus, the permanent appearance of the HSFL nanostructure at the bottom of the ablated microgroove (see Figure 3i) confirms that this type of LIPSS is formed after the metallic transition of the irradiated surface. The origin of the formation of nano-grooves (HSFL) is discussed in the following sections.

To study the quality of the LIPSS formed on the surface of the diamond, the SEM images were analyzed by 2D-FFT, as shown in Figure 3. The spatial volume dimensions such as periodicity and height of the nano-walls are the most important characteristics determining the quality of nanostructure. Figure 3j–l represent 2D-FFT of SEM images corresponding to Figure 3a–c. Figure 3m–o represent a cross section horizontal profile of Figure 3j–l. Note that the frequency peaks appearing around the center indicates the existence of a periodic nanostructure. It can be observed that the intensity of the peaks decreases with increase in the number of pulses. Thus, on the basis of this analysis we have classified the regions mentioned in Figure 2, where for instance Figure 3m,n indicates the existence of HSFL nanostructure due to visible peaks, while Figure 3o can be considered to be almost damaged due to weaker peaks. In addition, the reduction in the intensity of the peaks with the number of pulses indicates a reduction in the quality of the morphology of the nanostructure due to the thermal accumulation effect.

Based on this analysis, we also measured the nanostructure periods of region (2) mentioned in red **X** in Figure 2, where we estimated the peaks as Lorentzian function as indicated in Figure 3m. For instance, the average peaks projection value in Figure 3m is 4.774 ± 0.11 ${\mu m}^{-1}$ that corresponds to 209 ± 5 nm. 2D-FFT provides a very effective quantitative analysis tool and gives more representative results, either to determine the LIPSS periods, or to deduce the quality of the induced LIPSS.

**Figure 4:**
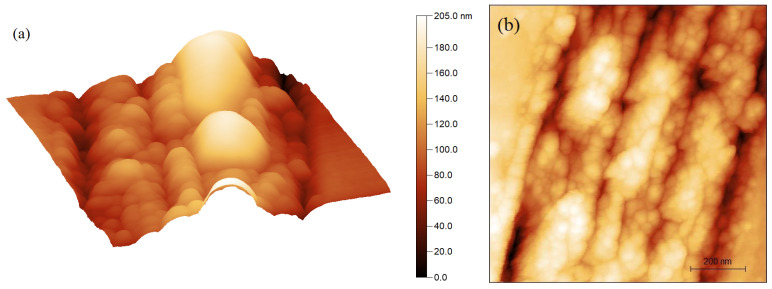
3D view of top LIPSS shown in Figure 3d showing the roughness of the nanowalls. (**a**) shows a side view taken after the etching as represented in top view in (**b**).

In order to probe the roughness of the nanowalls, which is the crest of the nano-gratings of the LIPSS, a magnified image of Figure 3d is shown in Figure 4. A wet etching reported in [26] has been carried out to clean the samples and remove the possible graphitic contamination from by the laser micromachining process. The apparent roughness of the nanowalls of the ablated structure shown in Figure 4a is confirmed by the AFM image taken after the etching and shown in Figure 4b.

From initial observation, it appears that the morphology of top LIPPS is rough and we suggest that the phenomenon of disorder induced by ultrafast laser excitation is mainly responsible for the origin of this nanoroughness. As we indicated in the introduction, the major application of nanostructures of HSFL type on the surface of the diamond is for development of black diamond, where these nanostructure induces a significant variation of the optical and electronic properties [27]. It has been mentioned that the drastic increase in the optical absorption of black diamond is not only due to the formation of the pseudo-metallic layer (graphitic layer) on the surface of the diamond, but also due to the periodicity of the ordered nanostructure on the surface of the diamond which acts as a sub-wavelength diffraction grating [22]. In addition, we suggest that this roughness, which is of the nanometric order, is one of the parameters that can be responsible for the improvement of the optical performance of the black diamond surface.

#### 3.1.2. Effect of Laser Power

**Figure 5:**
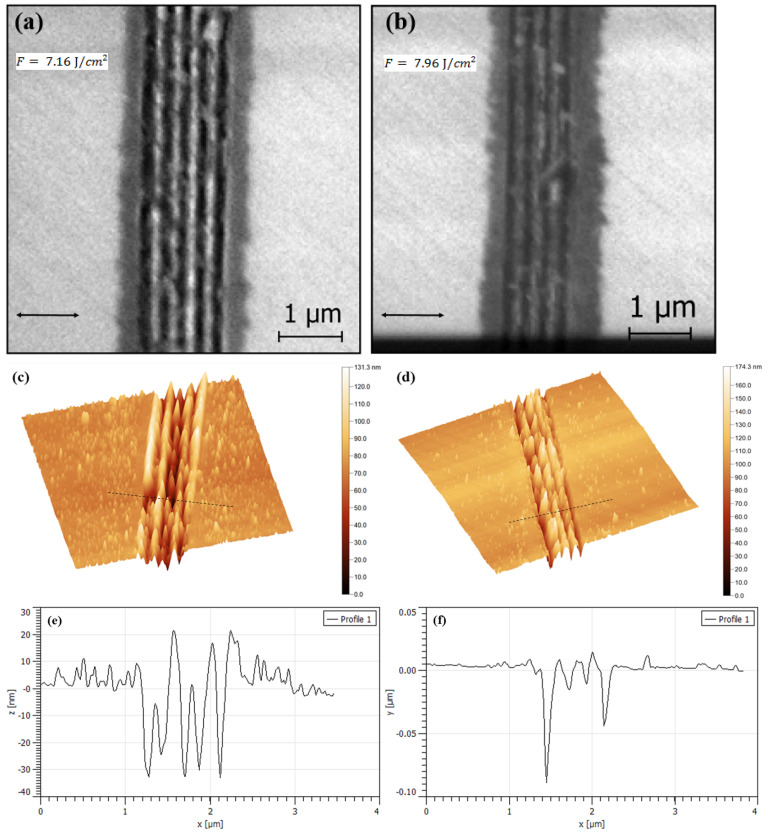
Representation of effect of laser fluence on the morphology of the diamond surface irradiated by 89 pulses (4.5 mm/s). (**a**,**b**) shows SEM images induced by 7.16 and $F=7.96\;J/{\mathrm{cm}}^{2}$, respectively. (**c**,**d**) AFM measurements of the (**a**,**b**), respectively (measured area 5 × 5 ${\mu m}^{2}$). (**e**,**f**) represent the cross-section profile corresponding to the lines marked in (**c**,**d**), respectively. The double-arrow shows the polarization of the incident laser beam.

Figure 5 shows the effect of laser power on the morphology of micro-nanogrooves induced by multipulse fs-laser on a diamond surface when the laser power is increased from 18 to 20 mW at fixed scan speed of 4.5 mm/s. Comparing Figure 5a,c,e formed under *P* = 18 mW with Figure 5b,d,f formed under *P* = 20 mW. With increasing laser energy, the height of LIPSS decreases and the ablated depth increases by close 100 nm. Therefore, we deduce that the laser power enhance the thermal accumulation effect which directly affects the LIPSS morphology.

It is also demonstrated by our plasmonic model below that 100 nm of depth is the maximum thickness of the graphite layer that can be created by laser irradiation.

#### 3.1.3. Periodicity of Nanostructures and Their Dependence

**Figure 6:**
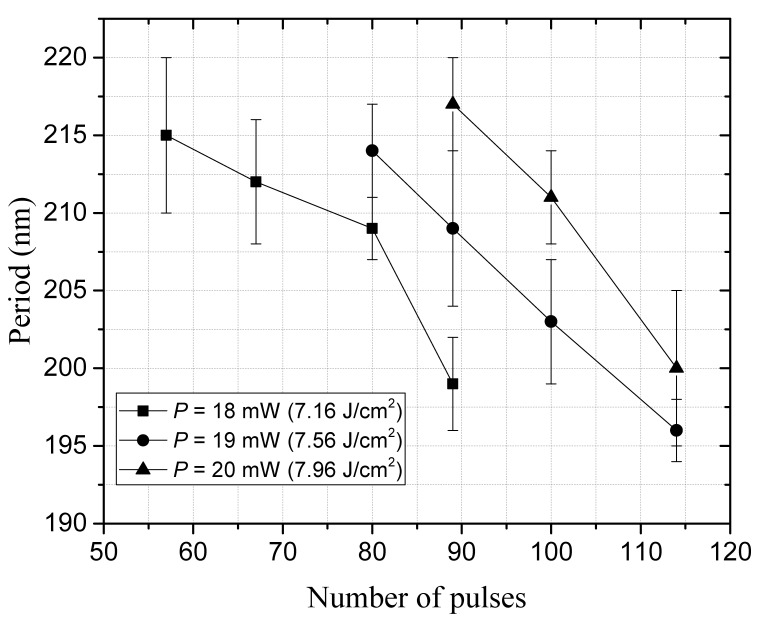
Evolution of periodicity of HSFL nanostructure with pulse number and laser power at 1030 nm wavelength.

Figure 6 shows the periodicity of the laser induced nanostructure as a function of pulses number and the laser power. We found that the periods of the HSFL nanostructures decreased with the number of pulses within the range between 217 nm and 196 nm under the present experimental conditions. We have also observed that the period of the nanostructure written increases with laser fluence when scanning perpendicular with respect to the laser polarization which is opposite trend for polarization parallel to the scanning direction in our previous work [10]. We suggest that the scanning direction has a significant effect on the periodicity of the nanostructures.

Note that the observed spatial range of periodic nanostructures 196–217 nm is narrow because of our experimental condition such as the fluence chosen around the ablation threshold, while it is possible to expand up to ∼λ/2 by decreasing the fluence below the melting threshold and increasing the number of pulses as demonstrated by Miyazaki et al. [28]. In this way, we enhance the multiphoton ionization which is the fundamental process of the disorder phenomenon and minimize the effect of thermal accumulation to avoid damaging the surface structures.

### 3.2. Theoretical Results

#### 3.2.1. Generalized Plasmonic Model

We have modeled employing generalized plasmonic model to understand the optical change of the diamond surface during the excitation of electron plasma induced by multipulse fs-laser and calculate the periods of nanostructure observed on the surface at a wavelength of 1030 nm. We consider air as the dielectric environment. The application of this model to semiconductors and to dielectric materials such as diamond is justified by the fact that a thin layer, which has a metallic character is induced during a multipulse femtosecond laser irradiation [29] known under the name of “Pseudo-metallic layer”. For this reason, excitation of SPP is used to calculate and explain the formation of periodic nanostructures in diamond.

The SPP dispersion relation is [30]:

(1) $$\begin{array}{c} k_{sp}=\frac{\omega }{c}{\left(\frac{\varepsilon_{pm}\varepsilon_{d}}{\varepsilon_{pm}+\varepsilon_{d}}\right)}^{\frac{1}{2}}. \end{array}$$

where ω is laser frequency, *c* is the speed of light in vacuum, $\varepsilon_{pm}$ is the dielectric function of pseudo-metal layer, $\varepsilon_{d}$ is the dielectric constant of the surrounding material medium and $k_{sp}=k_{sp1}+ik_{sp2}$ is the plasmon propagation number with the real part $ℜ\left(k_{sp}\right)\;=\;k_{sp1}=2\pi /\lambda_{sp}=\pi /\Lambda$ and the imaginary part $ℑ\left(k_{sp}\right)=k_{sp2}$, where $\lambda_{sp}$ is the plasmon wavelength and Λ is the period of nanostructures. The dielectric constant is approximated as $\varepsilon_{d}=m\varepsilon_{di}+\left(1-m\right)\varepsilon_{air}$, where *m* takes the value 0 at air/pseudo-metal interface and 1 at pseudo-metal/diamond interface, $\varepsilon_{air}$ dielectric constant of air and $\varepsilon_{di}=n^{2}$ dielectric constant of diamond at 1030 nm according to the relationship: $n^{2}=1+\frac{0.3306\lambda^{2}}{\lambda^{2}-175^{2}}+\frac{4.3356\lambda^{2}}{\lambda^{2}-106^{2}}$ (λ in nm ) [31].

The dielectric function of the pseudo-metal layer is given by the Drude model [32]:

(2) $$\varepsilon_{pm}=\varepsilon_{pm1}+i\varepsilon_{pm2}=1+\left(\varepsilon_{di}-1\right)\left(1-\frac{n_{e}}{n_{0}}\right)-\frac{\omega_{p}^{2}}{\omega^{2}}\frac{1}{1+\frac{i}{\omega \tau_{ee}}}.$$

with $\varepsilon_{pm1}=1+\left(\varepsilon_{di}-1\right)\left(1-\frac{n_{e}}{n_{0}}\right)-\frac{\omega_{p}^{2}}{\omega^{2}}\frac{1}{1+\frac{1}{\omega^{2}\tau_{ee}^{2}}}$ and $\varepsilon_{pm2}=\frac{1}{\omega \tau_{ee}}\frac{\omega_{p}^{2}}{\omega^{2}}\frac{1}{1+\frac{1}{\omega^{2}\tau_{ee}^{2}}}$ where $n_{e}$ electron plasma density excited during irradiation, $n_{0}$ is the electron concentration in the valence band, $\tau_{ee}$ is the electron-electron collision time, $\omega_{p}=\sqrt{\frac{n_{e}e^{2}}{\varepsilon_{0}m_{opt}^{*}m_{e}}}$ is the plasma frequency, where $m_{opt}^{*}$ the optical effective mass for our single crystal CVD diamond [10]. The value of parameters used are mentioned in Table 1.

By combining Equations (1) and (2), we have obtained the following generalized plasmonic model:

(3) $$\begin{array}{c} k_{sp1}=\frac{1}{\sqrt{2}}\frac{\omega }{c}{\left[\frac{\gamma \alpha }{\left(\alpha^{2}+\beta^{2}\right)}+\frac{\gamma }{{\left(\alpha^{2}+\beta^{2}\right)}^{\frac{1}{2}}}\right]}^{\frac{1}{2}}. \end{array}$$

(4) $$\begin{array}{c} k_{sp2}=\frac{\omega^{2}}{2c^{2}k_{sp1}}\frac{\gamma \beta }{\left(\alpha^{2}+\beta^{2}\right)}. \end{array}$$

where $\alpha =\varepsilon_{pm1}^{2}+\varepsilon_{pm2}^{2}+\varepsilon_{d}\varepsilon_{pm1},\;\beta =\varepsilon_{d}\varepsilon_{pm2}$ and $\gamma =\varepsilon_{d}\left(\varepsilon_{pm1}^{2}+\varepsilon_{pm2}^{2}\right)$

For more details see our previous work in Ref. [10]. This model was implemented using MATLAB software.

#### 3.2.2. Dielectric to Pseudo-Metallic Change

**Figure 7:**
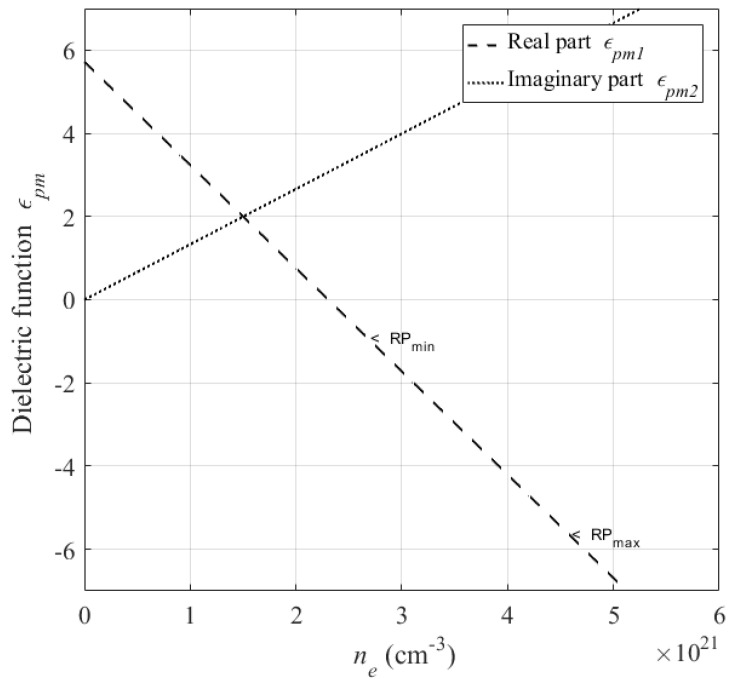
Dielectric function of diamond in function to electron plasma excitation by fs-laser at 1030 nm wavelength. ${\mathrm{RP}}_{\min}$ and ${\mathrm{RP}}_{\max}$ represent the resonance plasmonic in pseudo-metal/air and pseudo-metal/diamond interface, respectively.

Figure 7 shows the variation of diamond dielectric function as a function of the electron plasma density excited during irradiation of the surface by multipulse fs-laser. We see in Figure 7 that the real part of dielectric function decreased and takes on negative values with the increase of the electron density in the conduction band. We deduce that in the case of laser irradiation with ultra-short pulses, the dielectric function of diamond is modified due to the presence of free electron plasma induced by multi-photoionization where the dielectric material transforms into a transient metallic state called pseudo-metal layer.

This modification of the dielectric function directly affects the optical properties of the excited surface such as the reflectivity *R* and the optical penetration depth δ, with: $R=\frac{{\left(n_{r}-1\right)}^{2}+k^{2}}{{\left(n_{r}+1\right)}^{2}+k^{2}}$ and $\delta =\frac{c}{2\omega k}$, $n_{r}=\frac{1}{\sqrt{2}}{\left[\varepsilon_{pm1}+{\left(\varepsilon_{pm1}^{2}+\varepsilon_{pm2}^{2}\right)}^{\frac{1}{2}}\right]}^{\frac{1}{2}}$ is the real part of the refractive index and $k=\frac{\varepsilon_{pm2}}{2n_{r}}$ is the absorption coefficient [33].

**Figure 8:**
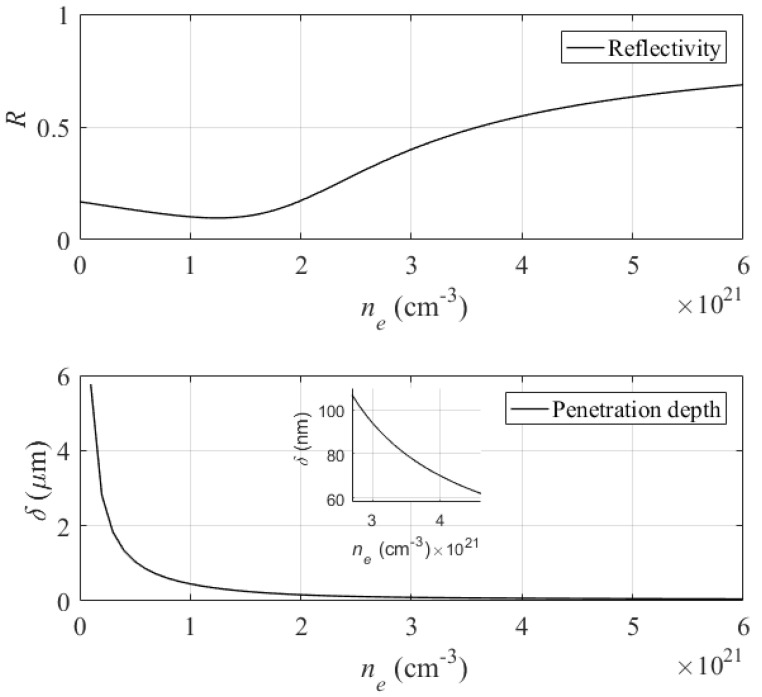
(**top**) reflectivity and (**bottom**) laser penetration depth of diamond in function of electron plasma excitation. The penetration depth corresponding to plasmonic excitation range is shown in the inset.

Figure 8 shows the evolution of reflectivity and laser penetration depth during irradiation by multipulse fs-laser. It is observed that the reflectivity is increased considerably with the increase in excited electron plasma. This augmentation shows that the excited surface takes on a metallic characteristic after excitation by the multipulse fs-laser. We deduce that the irradiation of a transparent material by multipulse fs-laser can lead to the formation of a pseudo-metal layer at its excited surface. The formation of this pseudo-metal layer has been demonstrated by the pump-probe technique [28].

Figure 8 also shows that laser penetration depth is reduced exponentially as a function of electron plasma excited, which confirms that the excited surface has taken on a metallic characteristic.

#### 3.2.3. Calculation of Periodic Nanostructure

**Figure 9:**
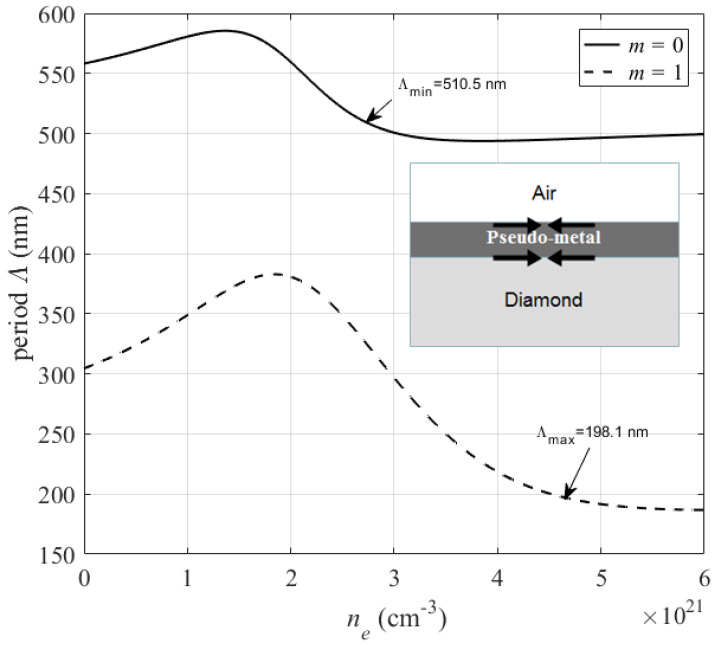
Calculation of LIPSS period evolution as a function of electron plasma excitation at pseudo-metal/air interface (m=0) and pseudo-metal/diamond interface (m=1) under fs-laser irradiation at 1030 nm wavelength. Optical system modeled as a pseudo-metal layer surrounded by the original material (diamond) and air is shown in the inset.

We have considered the optical system modeled as a pseudo-metal layer surrounded by the original material (diamond) and air, as shown within Figure 9. Figure 9 shows the evolution of the periodicity of HSFL-type nanostructures as a function of excited electron plasma. With the increase of electron plasma excited towards the conduction band, the dielectric function of the pseudo-metal layer decreases, and when it becomes $\varepsilon_{pm1}=-\varepsilon_{air}$ (m=0), a surface plasmon polariton can be excited at the pseudo-metal/air interface here called minimum plasmon resonance ${\mathrm{RP}}_{\min}$ (see Figure 7).

The RP occurs when the dielectric permittivity of the pseudo-metal and the dielectric surrounding medium is of the same value but of opposite sign, which thus produces a pole in the dispersion relation, what is called the asymptotic limit of the resonance frequency of the surface plasmon [34].

This initiation of plasmon excitation leads to the formation of the first ordered nanostructure with a period $\Lambda_{\min}=510.5$ nm as indicated in Figure 9. With increase in electron density in conduction band, the dielectric function of excited layer continues decreasing until reaching $\varepsilon_{pm1}=-\varepsilon_{di}$ (m=1) where a surface plasmon polariton maximal can be excited ${\mathrm{RP}}_{\max}$ (see Figure 9). This implies a construction of the last order nanostructure can be observed with period $\Lambda_{\max}=198.1$ nm, also called the period of saturation [35].

We showed previously [36] that $\Lambda_{\min}$ is slightly below λ/2 for several dielectric material tested by this model such as ${\mathrm{SiO}}_{2}$, ${\mathrm{Al}}_{2}O_{3}$, ZnO and AlAs, while $\Lambda_{\max}$ significantly decreases with bulk refractive index. Our results show that ordered nanostructure of HSFL type can be observed in plasmonic excitation range with period between $\Lambda_{\min}$ and $\Lambda_{\max}$. The periods of the nanostructures observed in our experiments is within this theoretically modeled plasmonic range. In addition, the pseudo-metallic layer formed during the irradiation can be estimated by plasmonic excitation range where it takes a thickness between 105 nm and 60 nm as shown in the inset of Figure 9. These calculated results are in good agreement with the experimentally depth measured above.

On the other hand, it is observed that the nanograting period decreases until a minimum value of 198 nm. This is significantly smaller than the estimate based on the second harmonic generation model, which gives a period of λ/2n=214 nm for 1030 nm where n=2.4 is the bulk refractive index of diamond. Then, by contrary reasoning, in order to take into account a period of 198 nm, a refractive index of 2.6 would be necessary, which reinforces the idea of plasmonic excitation on a layer having metallic characteristics formed during the irradiation by multipulse fs-laser on diamond surface.

### 3.3. Origin of HSFL Nanostructure

We provide a physical description showing the fundamental origin of the formation of HSFL type nanostructures perpendicular to the polarization of the laser incident on the diamond surface. This description may be valid for all dielectric and semiconductor materials. At picosecond duration or longer, the energy of the electrons excited by the laser is transferred to the bulk via electron–phonon collisions, which leads to a completely thermal solid-liquid melting transition. At the sub-picosecond scale, in particular the femtosecond duration, experimental [37] and theoretical [38] studies show that the covalent bonds of transparent materials become unstable when about 10% of the valence electrons are excited towards the conduction band. This instability then causes a very rapid disturbance of the bulk because the ions move randomly due to the generation of repulsive forces between them in the presence of plasma of hot electrons to seek a new position of electrostatic equilibrium. This means that excitation with ultrashort pulses causes an ultrafast material disorder leading to the formation of a cold liquid state, this mechanism called non-thermal melting (disorder). The diamond surface could therefore take on a metallic character layer while the bulk is still cold. This layer has a graphitic crystallography [39]. Recently, the cross-section of a laser-irradiated surface cut by an ion beam was observed by scanning electron microscopy, clearly showing an opaque layer induced on the surface, which we refer to here as the pseudo-metallic layer [40].

Miyaji et al. [29] and Richter et al. [35] showed that the ordered nanostructure is preceded by random nanostructures as a function of increase in the number of pulses. It has been suggested that the random nanostructure is formed by nano-ablation induced by a local field created on a rough surface [29]. This roughness of the surface comes back to the morphological change of the surface during non-thermal melting (disorder of the material). An intense local field could then form thanks to the non-uniform distribution of electrons excited by a Gaussian profile.

Micro-Raman spectra indicate that the material between the nano-grooves maintains the original composition of the diamond after femtosecond laser irradiation [41]. Thus, when the localized field reaches the ablation threshold, the nanoablation will be produced via Coulomb Explosions (CE) process on the nanometric scale and not by thermal explosion [17].

The physics of CE implies an ionic acceleration in the electrostatic field due to the separation of the charges produced by energetic electrons which escape from the target. This electric field is strong due to the separation of charges with lattice ions [25]. We deduce that the localized nanometric field plays the essential role in the ultrafast ablation process to form the random nanostructures. The nanoablation induced by local field improves the surface roughness and becomes a dispersive medium. This dispersive medium allows the incident *E*-field to couple coherently with free electrons plasma to excite the SPP [18]. Hence, we propose that the formation of ordered nanostructures is due to ordered nanoablation which can be attributed to the excitation of SPP where SPP replace the local field and will support nanoablation.

Finally, we suggest that non-thermal melting (disorder) and plasmonic excitation effect can be considered as a fundamental process for the nature of periodic nanoablation as the origin of HSFL nanostructures.

**Figure 10:**
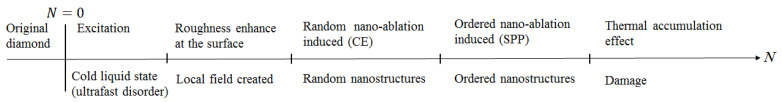
A proposed scenario for HSFL formation during multipulse femtosecond laser irradiation.

Figure 10 summarizes the formation of HSFL-type nanostructures on diamond surface during multipulse fs-laser irradiation.

## 4. Conclusions

In this work, HSFL-type nanostructures were fabricated on a diamond surface by laser pulses of 230 fs with a repetition rate of 250 kHz at a wavelength of 1030 nm. SEM characterization shows that LIPSS are perpendicular to the laser polarization. A 3D visualization of the microgrooves has shown that thermal accumulation effect reduces the spatial volume of LIPSS. A quantitative analysis by 2D-FFT makes it possible to study the number of pulse effect on the quality of LIPSS and to determine its periodicity. We observed also that the spatial morphology of top LIPSS walls is rough.

The optical development of the irradiated surface was followed and the periods of the nanostructures are calculated using the generalized plasmonic model. We observed a dramatic optical change with the increase in excited electron plasma. We deduce that the femtosecond laser can change the optical properties of diamond and form a pseudo-metallic layer on the irradiated surface, then a surface plasmon polariton can be excited on the pseudo-metal/dielectric surrounding medium interface. It is suggested that the nanoablation is initiated by the local field and improve perpendicular to the laser polarization by plasmonic excitation. So, we suggest that non-thermal melting and plasmonic excitation are the main processes responsible for the formation of HSFL-type nanostructures. We can confirm through our studies that the formation of nanostructures is not determined by the physical properties of materials in ordinary states, but rather we must consider the highly exciting states of surfaces for these problems. We expect more exciting discoveries on how to control the formation of nanostructures by plasmonic excitation.

## Figures and Tables

**Table 1:** The constants value used in this model.

Constant	$\varepsilon_{\mathrm{air}}$	$\varepsilon_{\mathrm{di}}$	$n_{0}$	$\tau_{\mathrm{ee}}$	$m_{\mathrm{opt}}^{*}$
Value	1	5.72	$10^{23}\;{\mathrm{cm}}^{-3}$	∼$10^{-15}$ s	0.3
